# Remarkable Role of Indoleamine 2,3-Dioxygenase and Tryptophan Metabolites in Infectious Diseases: Potential Role in Macrophage-Mediated Inflammatory Diseases

**DOI:** 10.1155/2013/391984

**Published:** 2013-02-18

**Authors:** Yuki Murakami, Masato Hoshi, Yukio Imamura, Yuko Arioka, Yasuko Yamamoto, Kuniaki Saito

**Affiliations:** ^1^Human Health Sciences, Graduate School of Medicine and Faculty of Medicine, Kyoto University, 53 Kawahara-cho, Shogoin, Sakyo-Ku, Kyoto 606-8507, Japan; ^2^Faculty of Health Science, Suzuka University of Medical Science, Suzuka, Mie, Tsu 510-0293, Japan

## Abstract

Indoleamine 2,3-dioxygenase 1 (IDO1), the L-tryptophan-degrading enzyme, plays a key role in the immunomodulatory effects on several types of immune cells. Originally known for its regulatory function during pregnancy and chronic inflammation in tumorigenesis, the activity of IDO1 seems to modify the inflammatory state of infectious diseases. The pathophysiologic activity of L-tryptophan metabolites, kynurenines, is well recognized. Therefore, an understanding of the regulation of IDO1 and the subsequent biochemical reactions is essential for the design of therapeutic strategies in certain immune diseases. In this paper, current knowledge about the role of IDO1 and its metabolites during various infectious diseases is presented. Particularly, the regulation of type I interferons (IFNs) production via IDO1 in virus infection is discussed. This paper offers insights into new therapeutic strategies in the modulation of viral infection and several immune-related disorders.

## 1. Introduction

Inflammation is the physiological response of the body to harmful stimuli, such as injury, pathogens, damaged cells, or irritants. Inflammatory response can be either acute or chronic, which leads to pathology. The major function of innate immune cells is identification and recognition of the injurious and/or foreign substances causing the defense response. Macrophages are actively involved in all phases of inflammation, and their role as effector and regulatory cells is now widely recognized. Another interesting and important role of macrophages is their high level of specialization and tissue specificity. While all tissue-bound macrophages differentiate from circulating monocytes, they acquire distinct characteristics and functions locally due to their response profiles. One of the major factors for this diversity is the complexity of microbial load as well as tissue architecture. Thus, it is not a surprise that some of the most sophisticated interactions between the host and parasites also dictate the most evolved phenotypic characteristics of the macrophage.

Indoleamine 2,3-dioxygenase 1 (IDO1) has been identified as an enzyme endowed with powerful immunomodulatory effects, resulting from its enzymatic activity that leads to catabolism of the essential amino acid L-tryptophan (L-TRP) [1, 2]. This enzyme is expressed in epithelial cells, macrophages, and dendritic cells (DCs) induced by proinflammatory cytokines [3–5]. The initial observation suggesting the immunoregulatory role of IDO1 dates back to the finding that its inhibition by 1-methyl-DL-tryptophan (1-MT) during pregnancy would cause rejection of semiallogeneic, but not syngeneic, fetuses in mice [6]. A second observation expanding upon that initial finding was that IDO1 mediates a bidirectional flow of information between cytotoxic-T-lymphocyte-associated-antigen-4- (CTLA-4-) expressing T cells and accessory cells of the immune system; IDO1 activation in antigen-presenting cells (APCs) by CTLA-4 ligation of CD80/CD86 “counterreceptors” on those cells represents an important effector pathway for regulatory T (Treg) cells, to induce and maintain peripheral tolerance [7, 8]. Third, it was later found that, in T cells, the general control nonderepressing-2 (GCN2) protein kinase, with a putative binding site for free acyl-transfer RNAs (tRNAs), acts as a molecular sensor for intracellular TRP, participating in the integrated stress response (ISR) pathway, which controls cell growth and differentiation [9]. Finally, IDO1 was found to possess signaling activity in DCs, which are stably turned into regulatory DCs by its activation. Thus, IDO1 may contribute to long-term immune homeostasis and immune-related functions not only in pregnancy, but also in infectious, allergic, autoimmune, and chronic inflammatory diseases, as well as in transplantation and immune-escaping tumoral mechanisms [7, 10–12]. The aim of this paper is to summarize the current knowledge about the physiological role of IDO1 following certain immune-related disorders. Further, new therapeutic targets via regulation of IDO1 are discussed against macrophage-related inflammatory diseases.

## 2. Tryptophan and Its Degradation Pathways

**Figure 1:**
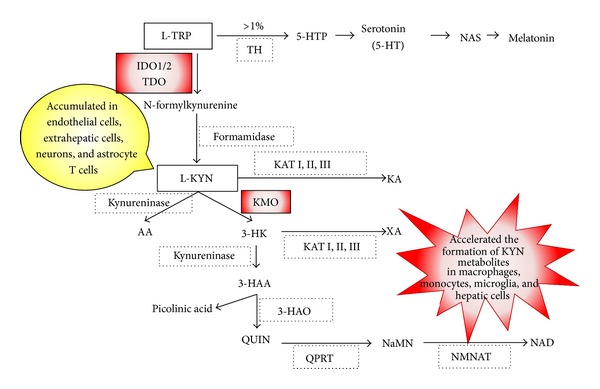
Schematic overview of the kynurenine pathway. It is estimated that only 1% of dietary tryptophan (TRP) can be converted into serotonin (5-HT). The remaining 99% of TRP is metabolized via the kynurenine (KYN) pathway. Tryptophan hydroxylase (TH), 5-hydroxy TRP (5-HTP), N-acetylserotonin (NAS), indoleamine 2,3-dioxygenase 1 and 2 (IDO1/2), tryptophan 2,3-dioxygenase (TDO), kynurenine 3-monooxygenase (KMO), kynurenine aminotransferase (KAT I, II, III), kynurenic acid (KA), anthranilic acid (AA), 3-hydroxykynurenine (3-HK), xanthurenic acid (XA), 3-hydroxyanthranilic acid (3-HAA), 3-hydroxyanthranilic acid oxidase (3-HAO), quinolinic acid (QUIN), quinolinic-acid phosphoribosyl transferase (QPRT), nicotinic acid mononucleotide (NaMN), nicotinamide mononucleotide adenylyltransferase (NMNAT), nicotinamide adenine dinucleotide (NAD).

TRP is an essential starting point of two biochemical pathways: (1) the enzyme tryptophan 5-hydroxylase converts TRP into 5-hydroxytryptophan, which is subsequently decarboxylated to 5-hydroxytryptamine (5-HT, serotonin), an essential neurotransmitter, and (2) two atoms of oxygen are inserted into TRP to form N-formylkynurenine, the first and rate-limiting step in the kynurenine (KYN) pathway (Figure 1). It is estimated that only 1% of dietary TRP can be converted into 5-HT [13]. The remaining 99% of TRP is metabolized via the KYN pathway. TRP is catalyzed by three different enzymes: tryptophan 2,3-dioxygenase (TDO), IDO1, and IDO2. While the expression and function of IDO2 have been well explored in the mouse model, there is a lack of knowledge about its expression and functional significance in human tissue. Human IDO1 and IDO2 seem to have different kinetic parameters and inhibition profiles. The Km for L-TRP of human IDO2 protein is approximately 500–1000-fold higher than that of mammalian IDO1 enzymes [14], and IDO2 is especially inhibited by 1-methyl-D-tryptophan (1-D-MT) [15, 16]. In contrast to both IDOs, TDO is a highly substrate-specific dioxygenase and deoxygenates only L-TRP and some TRP derivatives. The expression of TDO is normally restricted to mammalian liver cells where it is believed to regulate systemic TRP concentrations [17]. Although TDO has been identified in the brain and epididymis of some species recently [18], it has been found that TDO is expressed in human malignant glioma cells of the brain [19]. On the other hand, IDO1 is expressed in a broad variety of mammalian cells related to immune function, such as activated macrophages and DCs. IDO1 is induced by proinflammatory cytokines such as tumor necrosis-*α* (TNF-*α*) and IFN-*γ* [20]. The enzymatic activity of IDO1 is restricted to specific tissues, including lungs, cecum, colon, and epididymis [21]. In addition, Takikawa et al. found that immune activation, such as an endotoxin, lipopolysaccharide (LPS) injection, could induce IDO1 enzyme activity only in specific tissue; however, this local induction of TRP metabolism resulted in a threefold increase in KYN concentrations in serum [22]. Thus, these findings have suggested that TRP metabolism can be initiated in local tissues, whereas KYN may diffuse into the bloodstream. Therefore, increased KYN concentration in the serum can indicate increased TRP metabolism taking place in a specific tissue. While TDO and IDO1 alter local and systemic TRP concentrations and initiate the production of neuroactive and immunoregulatory TRP metabolites; the known immunologic function of TRP degradation is largely dependent on IDO1. In addition, the biological function of IDO2 is still unclear and needs clarification. Therefore, in this paper, we describe IDO1 and immune regulation, unless specifically noted.

## 3. Signal Pathways related to IDO1

IDO1 is induced by IFN-*γ*-mediated effects of the signal transducer and activator of transcription 1*α* (STAT1*α*) and interferon regulatory factor-1 (IRF-1). The IDO1 gene has two interferon-stimulated response elements (ISREs) and IFN-*γ*-activated site (GAS) element sequences in the 5′-flanking region [23–25]. IDO1 induction is also mediated by an IFN-*γ*-independent mechanism under certain circumstances [26–28]. Fujigaki et al. demonstrated that IDO1 induction by LPS is not mediated by STAT1*α* or IRF-1 binding activities that induce IDO1 transcriptional activity by IFN-*γ* in many cells [28]. LPS stimulation of human monocytes and macrophages activates several intracellular signaling pathways, including the IkappaB kinase-nuclear factor-*κ*B (NF-*κ*B) and mitogen-activated protein kinase (MAPK) pathways. These pathways, in turn, activate a variety of transcription factors that include NF-*κ*B and activator protein-1 (AP-1). A part of the induction of IDO1 by LPS is mediated by a signal from NF-*κ*B or p38-MAPK pathways. A homology search of the 5′-flanking region of the IDO1 gene shows consensus sequences for transcriptional factors such as AP-1, NF-*κ*B, and NF-IL-6, which are activated by LPS and other proinflammatory cytokines: TNF-*α*, IL-6, and IL-1*β*. Therefore, the IDO1 gene could be upregulated by LPS or these cytokines in a synergistic manner.

Posttranslational modifications (PTMs) of proteins perform crucial roles in the biological regulation of cells. PTMs provide a dynamic mechanism for regulating protein function and potentially change physical or chemical properties, activity, localization, or stability of proteins [29, 30]. Our group demonstrated for the first time that IDO1 activity is regulated by PTMs [31]. Peroxynitrite, a nitric-oxide- (NO-) derived reactive species, inhibits IDO1 activity via the nitration of tyrosine residues in IDO1 protein. This inhibition occurs at the posttranslational level because peroxynitrite inhibits IDO1 enzyme activity without affecting the expression level of the IDO1 protein. Activated macrophages can simultaneously generate large fluxes of NO and superoxide anions [32, 33], which rapidly combine to produce the far more reactive peroxynitrite anions. Peroxynitrite is considered to be produced by inflammatory cells to defend against infectious pathogens, such as parasites, viruses, and bacteria [34–36]. Thus, an understanding of protein nitration and PTMs on IDO1 will provide insight into the pathogenic mechanisms of inflammatory diseases related to macrophages and into novel therapeutic strategies for limiting tissue inflammatory injury.

## 4. Immune Regulation by IDO1

IDO1 was first isolated from rabbit intestine in 1967 [37], and it became rapidly clear that its induction serves the mechanism of antimicrobial resistance. Infection by bacteria, parasites, or viruses induces a strong IFN-*γ*-dependent inflammatory response. IFN-*γ*-induced IDO1 degrades TRP, and the depletion of TRP results in the regulation of intracellular pathogens [38–42]. On the other hand, Munn et al. provided evidence for a much broader immunoregulatory significance of TRP degradation by IDO1. They demonstrated that tolerance to allogeneic fetuses is regulated by IDO1-expressing cells in the mice placenta [1]. They and others also showed that a marked increase in IDO1 suppresses immune responses by locally depleting TRP and hence preventing T-lymphocyte proliferation using the IDO1 inhibitor, 1-MT [43–45]. These previous studies clearly showed that TRP degradation by IDO1 substantially contributes to immunoregulation, and therefore IDO1 has been considered as a strong immunoregulatory factor.

**Figure 2:**
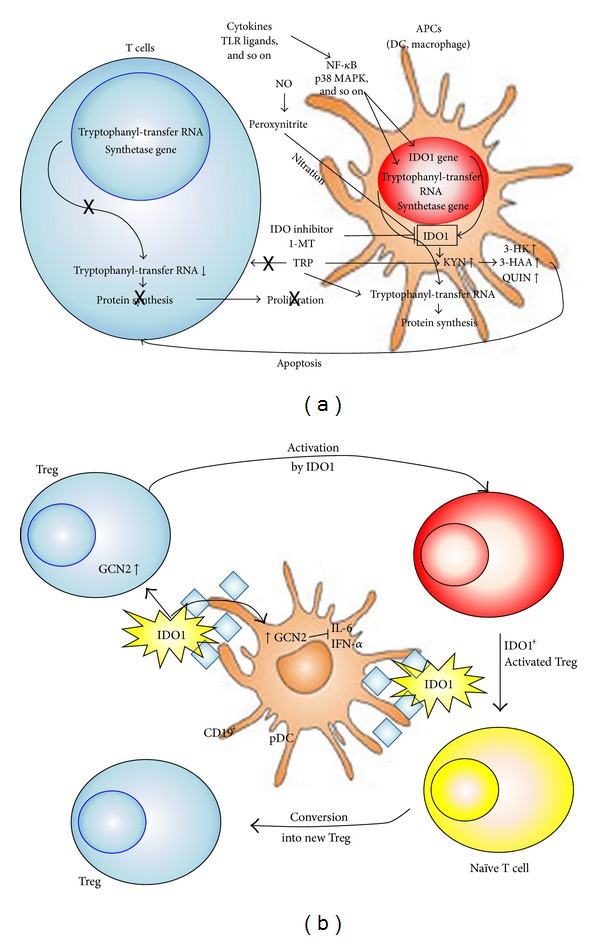
T-cell immune regulation by IDO1. (a) IDO1 is induced by IFN-*γ*-dependent and/or -independent signal pathways, depending on the variety of immune stimuli by macrophages and dendritic cells (DCs) [28, 83]. IDO1 activity is suppressed by the formation of NO or the competitive enzyme inhibitor, 1MT. Marked increases in IDO1 suppress immune responses by locally depleting L-TRP and preventing T-cell proliferation [44]. Expression of IDO1 has been observed in certain types of activated macrophages and DCs. IDO1-expressing cells deplete TRP from the extracellular milieu and secrete TRP metabolites, including KYN, 3-HK, 3-HAA, and QUIN, which induce T-cell apoptosis and suppress immune responses *in vitro*. (b) CD19^+^ plasmacytoid DCs (pDCs) express high levels of IDO1, which can activate mature regulatory T (Treg) cells via activation of the protein kinase general control nonderepressing-2 (GCN2) pathway of protein synthesis inhibition [84]. pDC-produced IDO1 and activated Treg can convert naïve T cells into new Treg. IDO1 acts in an autocrine manner to suppress pDC production of IL-6, which prevents the conversion of Treg into IL-17-producing Th17 proinflammatory cells [79]. IDO1 also downregulates type I IFN (IFN-*α*) production by pDC [80].

As shown in Figure 2, IDO1 is predominantly expressed in APCs of the immune system—the DCs, the monocytes, and the macrophages (Figure 2(a)) [46, 47]. IDO1 can be introduced by soluble cytokines such as IFN-*γ*, type I IFNs, transforming growth factor-*β* (TGF-*β*), TNF-*α*, or Toll-like receptors (TLRs) ligands such as LPS [27]. In addition, KYN and 3-hydroxykynurenine (3-HK) could be also involved in exacerbation of TRP starvation in T cells. Kaper et al. have proposed the existence of a positive feedback between IDO1-mediated TRP metabolism in DCs and KYN-induced TRP depletion in CD98-expressing T cells [48]. CD98 is expressed on astrocytes and activated T cells. T cells are sensitive to low levels of TRP and TRP metabolites* in vitro*. TRP deficiency specifically activates the GCN2 kinase in murine and human T cells, which leads to a halt in the G2 phase of T-cell division and T-cell suppression (Figure 2(b)) [49]. Moreover, a specific combination of TRP metabolites can inhibit anti-CD3 antibody-induced T-cell proliferation and induce T-cell apoptosis *in vitro* [50, 51]. The combination of low TRP concentrations and specific TRP metabolites leads to the generation of Tregs from naïve T cells* in vitro* [52, 53]. Tregs inhibit the activation, differentiation, and survival of effector T cells through the induction of IDO1 in APCs by ligation of inhibitory ligands and cytokines from Tregs [54].

**Figure 3:**
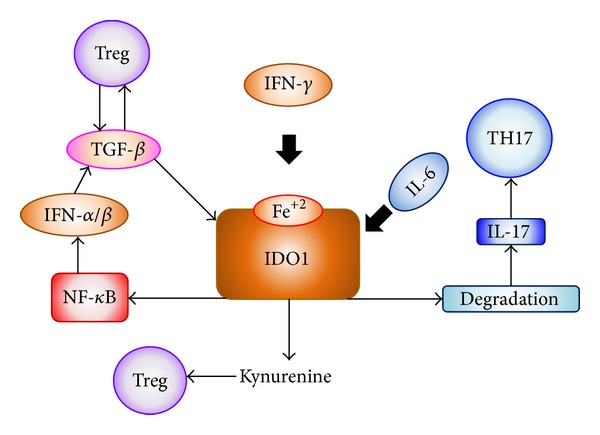
IDO1 induction and proinflammatory cytokines. IFN-*γ* drives intense enzymatic IDO1 activity, resulting in TRP depletion and high-level production of immunoregulatory TRP metabolites, KYNs, which may foster Tregs expansion. Induced Tregs use TGF-*β* to maintain an IDO1-dependent regulatory environment, with IDO1 mostly functioning as a signaling molecule. Both mechanisms are interrupted by IL-6, which drives IDO1 degradation as potent inflammatory stimuli enter the local environment [85].

It is possibly the selective pressure by Tregs that drove the evolution of the IDO1 mechanism from one operating in innate and inflammatory responses to pathogens [55, 56] to an effector mechanism of Treg function [57, 58]. Functional plasticity in DCs allows these cells to present antigens in an immunogenic or tolerogenic fashion, largely contingent on environmental factors [59]. Costimulatory and coinhibitory interactions between DCs and T cells are pivotal in tipping the balance between immunity and tolerance in favor of either outcome. When CD80/CD86 molecules on DCs were engaged to T cells, CTLA-4 (widely expressed by Tregs) was later shown to behave as an activating ligand for CD80/CD86 receptors, resulting in intracellular signaling events. Through an unidentified signal cascade, DCs release type I and type II IFNs that act in an autocrine and paracrine fashion to induce strong IDO1 expression and function [60]. KYN-dependent T-cell differentiation would contribute to expand the pool of Tregs [8]. However, in the long-term control of immune homeostasis and tolerance to self, IDO1 relies on different regulatory stimuli and cytokines, providing a basal function amenable to regulation by abrupt environmental changes (Figure 3) [61].

In a TGF-*β*-dominated environment and in the absence of IL-6, IDO1 activates a variety of downstream signaling effectors that sustain TGF-*β* production, production of type I IFNs, and a bias of plasmacytoid DCs (pDCs) toward a regulatory phenotype [62, 63]. IDO1 enhances its own expression and stably tips the balance between proinflammatory and anti-inflammatory NF-*κ*B activation.

## 5. Pathophysiologic Significance of Kynurenine Pathway Metabolites

IDO1-expressed DCs are able to lower TRP concentration, increase KYN concentration, and suppress the allogeneic T-cell response [50]. The TRP metabolites KYN, 3-HK, and 3-hydroxyanthranilic acid (3-HAA) inhibit T-cell proliferation by a time-dependent cytotoxic action, an effect which concerns mainly not only the activated T cells, but also B and natural killer (NK) cells. It has also been reported that KYN was able to reduce proliferation of human peripheral blood lymphocytes (PBL)* in vitro* [64]. The cytotoxic action of 3-HK can be attributed to the production of hydrogen peroxides which results in the damaging action of free hydroxyl radical [65]. As with KYN, when 3-HK was administrated exogenously, it effectively reduced symptoms in allergic inflammation [66]. The toxic action of 3-HAA is more complex. Although the final effect of 3-HAA results in the cell death of T cells, thymocytes [51] and monocyte-derived macrophages [67], the mechanisms involved in the cell death, might depend on the cell type. The formation of cytotoxic-free hydroxyl radical may be involved in 3-HAA-induced cell death in monocyte-derived macrophages [67]. L-KYN is considered to be the end product of KYN pathway metabolism in most extrahepatic cells, whereas macrophages produce the largest amount of quinolinic acid (QUIN) in accordance with the highest activities of kynurenine 3-monooxygenase (KMO) and kynureninase [4, 68]. In fact, Heyes et al. showed that macrophages stimulated with IFN-*γ* may be an important source of accelerated TRP conversion into KYN metabolites in inflammatory diseases [69]. Further, they showed that increased activities of KYN pathway enzymes, including IDO1 and KMO following systemic immune stimulation and HIV infection, in conjunction with macrophage infiltration, resulted in acceleration of the local formation of KYN metabolites, especially QUIN [21, 70]. Therefore, KMO is considered as a secondary regulatory enzyme for the KYN pathway, and macrophages, including monocytes, play a key role in the production of KYN metabolites.

## 6. Type I IFNs Production and IDO1

**Figure 4:**
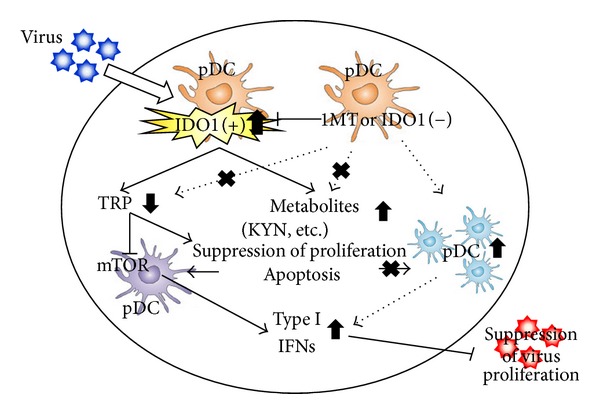
The mechanism of IDO1 regulation on viral infectious diseases. Innate defense occurs when pathogens contact or invade host cells and elicit the production of cytokines and chemokines, which in turn induce an influx of immune cells that affect pathogen clearance. Type I IFNs are critical mediators of innate immunity and limit disease caused by many viruses [73, 86]. The enhancement of TRP breakdown by IDO1 regulates the signal pathway for IFNs production, and TRP metabolites might contribute to the function of IFNs producing cells.

IDO1 activities in various tissues are induced by several cytokines after viral infection. However, the role of IDO1* in vivo* after parasitic or viral infection is not fully understood. Recently, our group demonstrated that inhibition of increased IDO1 activity attenuates *Toxoplasma gondii* replication in the lung, and the inflammatory damage is significantly decreased by the administration of the IDO1 inhibitor after infection [71]. Some *in vitro* studies indicated that IFN-*γ*-induced antitoxoplasma activities are involved in IDO1-dependent mechanisms. These *in vitro* studies showed that IFN-*γ*-induced IDO1 degraded TRP in the culture medium, and the depletion of TRP resulted in the suppression of the growth of the parasites [39]. However, our experiments and the most recent study demonstrated that IDO1 ablation reduced local inflammation and parasite burdens, as did pharmacological inhibition of IDO1* in vivo* [72]. Although IDO1 is certainly not the only regulator that plays a role as an antimicrobial, these studies show that the lack of the IDO1 gene or the inhibition of increased IDO1 activity suppressed the parasites' replication *in vivo* and that TRP degradation and KYNs production are not the only mechanisms of host resistance to early infection with these parasites. On the other hand, Hoshi et al. investigated the role of IDO1 in chronic viral infection diseases in mice infected with LP-BM5 murine leukemia virus (MuLV), including both replication-competent and replication-defective viruses, which resulted in the development of a fatal immunodeficiency syndrome in mice, known as murine AIDS [73]. Murine AIDS is characterized by activation and proliferation of T and B cells, impaired T- and B-cell function, an aberrant regulation of cytokine production, hypergammaglobulinemia, decreased NK cell function, the development of B-cell lymphoma, and the susceptibility to opportunistic infections [74]. Hoshi et al. used IDO1 gene-deficient (IDO1 K.O.) mice and IDO inhibitor to examine whether IDO1 is an important factor for immune regulation against LP-BM5 infection and especially whether the presence of IDO1 is necessary for the induction of cytokines and IDO1-related molecules, which are important for viral clearance. Remarkably, they demonstrated that absence of IDO1 upregulated type I IFNs and downregulated virus replication in IDO1 K.O. mice with LP-BM5 infection [73]. Their finding is the first piece of evidence that the absence of IDO1 is involved in the clearance of murine retroviral infection via upregulated type I IFNs (Figure 4). Further, they also recently examined the roles of IDO1 in immune regulation in encephalomyocarditis virus (EMCV) infection by using IDO1 K.O. mice or the IDO inhibitor, 1-MT. EMCV, a member of the Picornaviridae family which includes the *Enterovirus* genus, can cause acute myocarditis in various animals. EMCV infection in mice is an established model for viral myocarditis, dilated cardiomyopathy, and congestive heart failure [75]. They demonstrated that type I IFNs are upregulated, resulting in suppressed EMCV replication by IDO1 knockdown or inhibition [76]. They also found that treatment of IDO1 K.O. mice with KYN metabolites eliminated the effects of IDO1 knockdown on the improved survival rates. These results suggested that KYN metabolites regulate the production of type I IFNs by decreasing the number of macrophages. Viruses initially activate the innate immune system, which recognizes viral components through pattern-recognition receptors (PRRs). Currently, three classes of PRRs have been shown to be involved in the recognition of virus-specific components in innate immune cells, which are TLRs, retinoic-acid-inducible-gene-I- (RIG-I-) like receptors (RLRs), and nucleotide-oligomerization-domain- (NOD-) like receptors (NLRs). Of these, TLRs and RLRs are especially important for the production of type I IFNs and various cytokines [77]. Therefore, these reports suggest that the enhancement of TRP breakdown by IDO1 regulates several signal pathways, which is related to IFNs production. TRP metabolites might contribute to the function of IFNs producing cells, like macrophages and DCs. The role of IDO1 may be complex; it may depend on the difference of disease stages (acute/chronic disease) and/or the stimulus pathogens. Kumagai et al. showed that lung infection with Newcastle disease virus (NDV) led to type I IFN, IFN-*α* production in alveolar macrophages, and conventional DCs (cDCs), but not in pDCs [78]. Specific depletion of macrophages caused a marked defect in initial viral elimination in the lung, and pDCs produced type I IFNs in the absence of macrophage-mediated viral recognition. These results suggest that pDCs work as immune regulators when the first defense line by macrophages is broken. Macrophages are important for the initial response to viral infection in the lung. Besides, a subpopulation of CD19^+^ pDCs produces high levels of the TRP-catabolizing enzyme IDO1 [44]. Production of IDO1 by pDC has been linked directly to activation of naturally occurring Foxp3^+^ Treg through modulation of the GCN2 pathway, which leads to inhibition of protein synthesis and Treg activation [63]. IDO1 plays a dual regulatory role by preventing conversion of these Tregs into proinflammatory Th17 cells through autocrine inhibition of IL-6 production via upregulation of GCN2 in pDC [79] and inhibited production of type I IFN and IFN-*α*, which may limit their ability for activating innate and adaptive antitumor immunity [80]. The mammalian target of rapamycin (mTOR) pathway is also reported in regulating type I IFNs production by pDCs [81]. Additionally, TRP breakdown by IDO1 may regulate mTOR inhibition pathway [82]. Therefore, the degradation of local TRP and increased TRP metabolites by activated IDO1 may stimulate several signal pathways and induce cell death, resulting in the inhibition of IFNs production.

## 7. Conclusions

IDO1 is not only pivotal in limiting potentially exaggerated inflammatory reactions in response to danger signals and in assisting the effector functions of Treg cells, but also an important component of the regulatory system that presides over long-term control of immune homeostasis. On the other hand, TRP metabolism via KYN pathway is a good example of how metabolism of small molecules can impact the immune system. Therefore, induction of the KYN pathway and/or controlling the systemic TRP concentrations by stimulation of immune cells or by diet might be an effective strategy for treatment of virus infection and immune diseases. In addition, understanding the subsequent steps on the KYN pathway and the physiological mechanisms responsible for regulation of KYN and concentration of its metabolites in biological fluids may be important for development of drugs in the future. We believe that further findings on the mechanism of immune regulation by IDO1 and TRP metabolites might contribute to the implementation of a novel therapy protocol, which would target several immune disorders.
